# Synthesis and Thermoreversible Gelation of Coil–Rod Copolymers with a Dendritic Polyethylene Core and Multiple Helical Poly(γ-benzyl-L-glutamate) Arms

**DOI:** 10.3390/polym15224351

**Published:** 2023-11-08

**Authors:** Yuliang Lu, Dongtao Liu, Xinjie Wei, Jiming Song, Qiaogang Xiao, Kezheng Du, Xinbo Shi, Haiyang Gao

**Affiliations:** 1 China National Offshore Oil Corporation Energy Technology & Services Limited Shenzhen Branch, Shenzhen 518067, China; luyl@cnooc.com.cn (Y.L.); liudt@cnooc.com.cn (D.L.); weixj6@cnooc.com.cn (X.W.); songjm2@cnooc.com.cn (J.S.); xiaoqg@cnooc.com.cn (Q.X.); dukzh@cnooc.com.cn (K.D.); 2 School of Materials Science and Engineering, PCFM Lab, GD HPPC Lab, Sun Yat-Sen University, Guangzhou 510275, China; bob@chainwalkingcn.com; 3 Chain Walking New Material Technology (Guangzhou) Co., Ltd., Guangzhou 511457, China

**Keywords:** dendritic polyethylene, poly(γ-benzyl-L-glutamate), fibers, gelation, gels

## Abstract

Coil–rod copolymers with a dendritic polyethylene (DPE) core and multiple helical poly(γ-benzyl-L-glutamate) (PBLG) arms (DPE-(PBLG)_n_) were prepared by palladium-catalyzed copolymerization in tandem with ring-opening polymerization (ROP). Macroinitiator (DPE–(NH_2_)_11_) was firstly prepared by the group transformation of DPE–(OH)_11_ generated from palladium-catalyzed copolymerization of ethylene and acrylate comonomer. Coil–helical DPE-(PBLG)_11_ copolymers were prepared by ROP of γ-benzyl-L-glutamate-N-carboxyanhydride (BLG-NCA). These DPE-(PBLG)_11_ copolymers could form thermoreversible gels in toluene solvent, and the dendritic topology of the DPE core increased the critical gelation concentrations. The self-assembled nanostructure of gels was fully characterized by transmission electron microscopy (TEM), atomic force microscopy (AFM), small-angle X-ray scattering (SAXS), and wide-angle X-ray diffraction (WAXD), and the morphology of the fibrous structure was a twisted flat ribbon through a self-assembled nanoribbon mechanism. The self-assembled fibers formed by DPE-(PBLG_45_)_11_ are more heterogeneous and ramified than previously observed fibers formed by PBLG homopolymer and block copolymers.

## 1. Introduction

As a class of polymers with unique chain architecture, dendritic polymers have received considerable attention [1]. Compared with linear, branched, and crosslinked polymers, dendritic polymers have compact globular shape featured with extensive branch-on-branch chain architectures, which renders them with unique physical properties such as dilute melt/solution viscosity, excellent solubility in solvent, and plenty of reactive terminal groups [2,3,4,5]. Dendritic polymers have shown good application potential in rheological additives, drug delivery, biomedical materials, and nanoelectronics [6,7,8]. 

The common synthesis of dendritic polymers through stepwise polymerization of polar AB_2_ or vinyl monomers offers structural control and uniformity, whereas the tedious and sophisticated multistep synthesis also limits their general applicability. Based on α-diimine nickel/palladium-catalyzed ethylene chain walking polymerization discovered by Brookhart [9,10], Guan has pioneeringly reported the synthesis of a hydrophobic hyperbranched/dendritic polyethylene (HBPE/DPE) using α-diimine palladium catalysts by turning reaction temperature and ethylene pressure [11,12,13]. The resultant polymeric product is a class of hyperbranched/dendritic polyethylene comprising multiple branches joined together, which is different from highly branched PE and low-density PE (LDPE). 

Because the PE is full nonpolar, the application of dendritic polyethylene (DPE) is often limited. Because α-diimine palladium catalysts display two striking features including chain walking and excellent tolerance toward polar comonomers, the palladium-catalyzed copolymerization of ethylene and polar comonomers opens a way to the development of functionalized DPEs tethered with various polar groups [14,15,16]. These functionalized groups are often positioned at the branching ends of DPE, which is a result of chain walking mechanism presented by Brookhart [10]. The exposition of reactive end groups on the periphery of DPE facilitates construction of functional copolymers for improving material properties and expanding applications. Several research groups including Guan, Ye, Xu, and our groups have prepared various HBPE/DPE copolymers with multiple functionalized arms by a tandem synthetic strategy combining palladium-catalyzed chain walking copolymerization with other living/controlled polymerization techniques [17,18,19,20,21,22,23,24,25,26,27,28,29]. Despite these contributions, functional polymer arms grafted on HBPE/DPE are only limited to coil polymer chain segments. No rod-like polymer block has been introduced on the HBPE/DPE to date, especially artificial polypeptide, although linear/branched PE-polypeptide copolymers have been reported to be biologically interesting [30,31,32,33,34,35,36,37,38].

Poly(γ-benzyl-L-glutamate) (PBLG) polymer is a common artificial polypeptide, which can assume a rigid α-helical conformation. Therefore, PBLG is usually used as a versatile building block for constructing copolymers with unique structures and investigating their phase behaviors [39]. Although the preparation and phase behaviors of PBLG-based block copolymers with rod–coil superstructures have been extensively studied [40,41,42,43,44,45], few copolymers with multiple PBLG arms are reported. In this contribution, we report the first synthesis of coil–rod copolymers with a DPE core and multiple helical PBLG arms by a tandem approach of α-diimine palladium-catalyzed copolymerization of ethylene and polar comonomer in combination with ROP of *γ*-benzyl-*L*-glutamate-*N*-carboxyanhydride (BLG-NCA). We can envision that the functional DPE-(PBLG)_n_ copolymers have a unique architecture with a coil DPE core and the multiple rod PBLG arms, which is very interesting for self-assembly. DPE-(PBLG)_n_ copolymers can self-assemble into fibrils to form thermoreversible organogels in toluene by a nanoribbon mechanism. Importantly, the dendritic topology of DPE core not only increases the critical gelation concentration, but also leads to heterogeneous and ramified fibers.

## 2. Materials and Methods

### 2.1. General Procedures

All manipulations involving air- and moisture-sensitive compounds were performed under a nitrogen atmosphere with standard anhydrous and anaerobic operation.

### 2.2. Materials

Dichloromethane were dried and purified from calcium hydride under a nitrogen atmosphere, and toluene and hexane from were dried and purified by sodium/potassium alloy under a nitrogen atmosphere. Ethylene gas (99.99%) was purchased from Guangzhou Gas company and purified by passing through columns packed with deoxygenation agent and molecular sieves before polymerization. N-*tert*-butoxycarbonyl-L-valine (BOC-L-valine) were purchased from Sigma-Aldrich and directly used without purification. The α-diimine palladium catalyst was synthesized according to the literature [10]. Other commercially available chemical reagents were purchased and used as received.

### 2.3. Synthesis of γ-Benzyl-L-glutamate-N-carboxyanhydride

γ-Benzyl-L-glutamate-*N*-carboxyanhydride (BLG-NCA) was prepared according to reported literature [38]. Some 15 g (0.063 mol) of γ-Benzyl-L-glutamate was dissolved in 450 mL of ethyl acetate solvent in a round-bottom flask fitted with a stirring bar under N_2_ atmosphere. After heating to reflux, 6.3 g (0.021 mol) of triphosgene was injected and the reaction kept stirring and refluxing under nitrogen atmosphere. After 5 h, the reaction was then stopped and cooled slowly to −5 °C. The resultant cold solution was transferred to a separatory funnel and washed with 200 mL of pure and cold water at 0 °C. The organic layer was then washed with 200 mL of NaHCO_3_ solution at 0 °C and then dried with anhydrous MgSO_4_. The clear solution was filtered and concentrated on a rotary evaporator. An equal volume of hexane was then added to induce crystallization of the NCA. Pure polymerization-grade BLG-NCA product was obtained by three times recrystallization from mixture solvent (hexane:ethyl acetate = 3:1). ^1^H NMR (CDCl_3_, ppm): 7.39–7.25 (m, 5H), 6.25 (s, 1H), 5.16 (s, 2H), 4.38 (t, 1H), 2.63 (t, 2H), 2.38–2.06 (m, 2H).

### 2.4. Synthesis of Polar Comonomer HEA-TMS

2-Hydroxyethyl acrylate (HEA) (0.1 mmol) was mixed with 0.12 mol imidazole in 20 mL CH_2_Cl_2_, and then mixture solution in CH_2_Cl_2_ was dopped into trimethylchlorosilane (TMS) solution in 20 mL CH_2_Cl_2_ at 0 °C. After addition, the reaction system was slowly heated to room temperature and stirred overnight. The resultant mixtures were filtrated to remove imidazole hydrochloride precipitation. The crude product trimethylsilyl-protected 2-hydroxyethyl acrylate (HEA-TMS) was generated from the filtrate by washing with water two times, drying by MgSO_4_, and evaporating solvent. HEA-TMS was further purified by column separation and was directly used in copolymerization. HEA-TMS was characterized by ^1^H NMR spectroscopy. ^1^H NMR (CDCl_3_, ppm): 4.48 (d, 2H), 6.23 (m, H), 5.85 (d, H), 4.24 (t, 2H), 3.79 (t, 2H).

### 2.5. Synthesis of DPE-(OH)_11_ by Palladium-Catalyzed Copolymerization of Ethylene and HEA-TMS

Palladium-catalyzed copolymerization of ethylene and the HEA-TMS comonomer was performed in a 300 mL Parr autoclave. The autoclave was firstly dried for 4 h at 150 °C, followed by cooling to room temperature under vacuum, and purged three times with ethylene. A prescribed (0.2 M) amount of HEA-TMS solution in CH_2_Cl_2_ was added into the autoclave. After stirring for 10 min at 35 °C, 3.0 mmol of α-diimine palladium catalyst in 10 mL CH_2_Cl_2_ was injected into the autoclave, and the total system volume was kept at 200 mL. Polymerization was carried out at 0.1 atm ethylene pressure. After 24 h, the polymerization was stopped by venting the autoclave and the oily product was precipitated out using methanol. To remove catalyst residues, the polymeric product was further purified by flash column separation packed with neutral alumina and silica gel using hexane as solvent. The solvent was subsequently evaporated to obtain colorless polymeric product. The deprotection of TMS was done by treating the copolymers with 10.0 equiv. tetrabutylammonium fluoride in 100 mL THF overnight to remove all the free hydroxyl groups. After concentration by rotary evaporation, the product was then obtained by precipitation by adding 300 mL methanol solvent and then drying under vacuum at 40 °C. The microstructure of the product was characterized by ^1^H NMR spectroscopy. ^1^H NMR (CDCl_3_, ppm): 4.20 (t, 2H); 3.72 (s, 2H); 2.35 (m, 2H), 1.4–0.75 (PE). The amount of HEA in copolymer was 0.32 mol% determined by^1^H NMR spectroscopy, *M_n_* of copolymer determined by gel permeation chromatography (GPC) with a light scattering (LS) detector (GPC-LS) was 95 kg/mol with PDI of 1.38, and branch density was 102/1000 C.

### 2.6. Synthesis of DPE-(NH_2_)_11_

First, 2.0 g DPE-(OH)_11_ was added into a dried flask, and then 0.2 g N-*tert*-butoxycarbonyl-L-valine (BOC-L-valine) solution in toluene (40 mL) was also injected. The solution was treated with 0.2 g *N,N*-dicyclohexylcarbodiimide (DCC) solution and then stirred at room temperature. After 72 h, the dicyclohexylurea was removed by filtration. Then, the polymer solution was poured into an excess of cold methanol and the resultant polymer (DPE-(NH-BOC)_11_) was precipitated and purified by filtrating, washing with methanol four times, and drying under vacuum at 30 °C. ^1^H NMR (CDCl_3_, ppm): 4.30 (t, 4H), 2.36 (s, 2H), 1.42 (s, 9H), 1.4–0.75 (PE and CH(CH_3_)_2_).

The DPE-(NH-BOC)_11_ solution in 30 mL toluene was treated with 15 mL of trifluoroacetic acid (TFA). After 2 h, TFA was then removed by rotary evaporation. The resultant product was re-dissolved in 50 mL toluene solvent, and then solution was extracted several times with aqueous NaHCO_3_ and water. The polymer was obtained by adding a large amount of methanol into its toluene solution. The precipitation was collected by filtration. The polymer (DPE-(NH_2_)_11_) was obtained by washing with methanol four times and drying in vacuum at room temperature. ^1^H (CDCl_3_, ppm): 4.35 (m, 4H), 2.34 (s, 2H), 1.4–0.75 (PE and CH(CH_3_)_2_). 

### 2.7. Synthesis of DPE-(PBLG)_11_ Copolymers

A dried round-bottom flask with a stirring bar was vented several times using N_2_. The appropriate amount of DPE-(NH_2_)_11_ macroinitiator in 5 mL dried CH_2_Cl_2_ was transferred into the round-bottom flask. The desired amount of BLG-NCA monomer solution in dried CH_2_Cl_2_ was introduced into the round-bottom flask, and the polymerization reaction was continuously stirring for 72 h at 25 °C. The copolymer solution in CH_2_Cl_2_ was poured into an excess of hexane, and then precipitations were collected by filtration. The copolymer was further purified by washing with hexane five times and drying under vacuum at room temperature. ^1^H NMR (CDCl_3_, ppm): 7.83 (d, 1H), 7.29–7.20 (m, 5H), 5.07 (s, 2H), 4.56 (s, 1H), 2.44 (t, 2H), 2.08–1.92 (m, 2H), 1.4–0.75 (PE). 

### 2.8. Gelation Experiment for DPE-(PBLG)_11_

In a 10 mL vial, the desired amount of DPE-(PBLG)_11_ copolymers were transferred in 3 mL of toluene solvent. The mixture was heated to 90 °C and stirred until the DPE-(PBLG)_11_ copolymers were fully dissolved in toluene. The solution was then cooled slowly. At the critical gelation temperature, the transparent organogels were formed and observed. The apparent critical gelation concentrations (*C*_gel_) and the critical gelation temperatures (*T*_gel_) were determined by the test tube inversion method.

### 2.9. Measurements

^1^H NMR spectra were performed on a Bruker 500 MHz instrument in CDCl_3_. Fourier transform infrared (FTIR) spectra of polymers were determined on a Nicolet NEXUS–670 FTIR spectrometer. Gel permeation chromatography (GPC) analysis of DPE was conducted on a Waters Breeze instrument system using THF as the eluent with a flow rate of 1.0 mL/min at 40 °C. The instrument was equipped with a differential refractive index (DRI) detector and a light scattering (LS) detector (λ = 670 nm) to detect absolute molecular weight and distribution (*M_w_*/*M_n_*). Differential scanning calorimetry (DSC) analysis of copolymer samples was conducted with a Perkin Elmer DSC-8000 instrument. The second heating curves were recorded from 30 °C to 150 °C with a heating and cooling rate of 10 °C/min. Transmission electron microscopy (TEM) observation of samples was taken on a TEM instrument (Philips TECNAI) (voltage = 120 kV). Atomic force microscopy (AFM) morphological observation of the gels was carried out on SPA300 HV Probe station (Tokyo, Japan) in tapping mode. Small-angle X-ray scattering (SAXS) measurement was performed on a Bruker Nanostar U system with a Cu Ka radiation (λ = 1.54 Å) operating at 40 kV and 35 mA. The scattered results were collected using a 2D photon counting HI-STAR detector in a *q* range from 0.015 to 0.2 Å. The *d*-spacing data was calculated from the formula *d* = 2π/*q*. Wide X-ray diffraction (WAXD) results were collected on a Rigaku RINT-2000 counter diffractometer with a Cu Ka radiation source operating at 40 kV and 30 mA. For X-ray experiments, gels were slowly dried under air for a long duration.

## 3. Results and Discussion

### 3.1. Synthesis and Characterization of DPE Tethered with Amine Groups

**Scheme 1:**
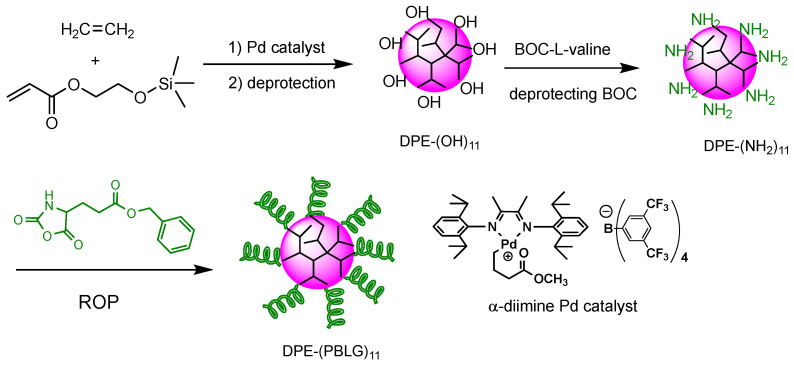
Synthetic route to DPE-(PBLG)_n_ by combination of chain walking copolymerization of ethylene with HEA-TMS and ROP of BLG-NCA.

**Figure 1:**
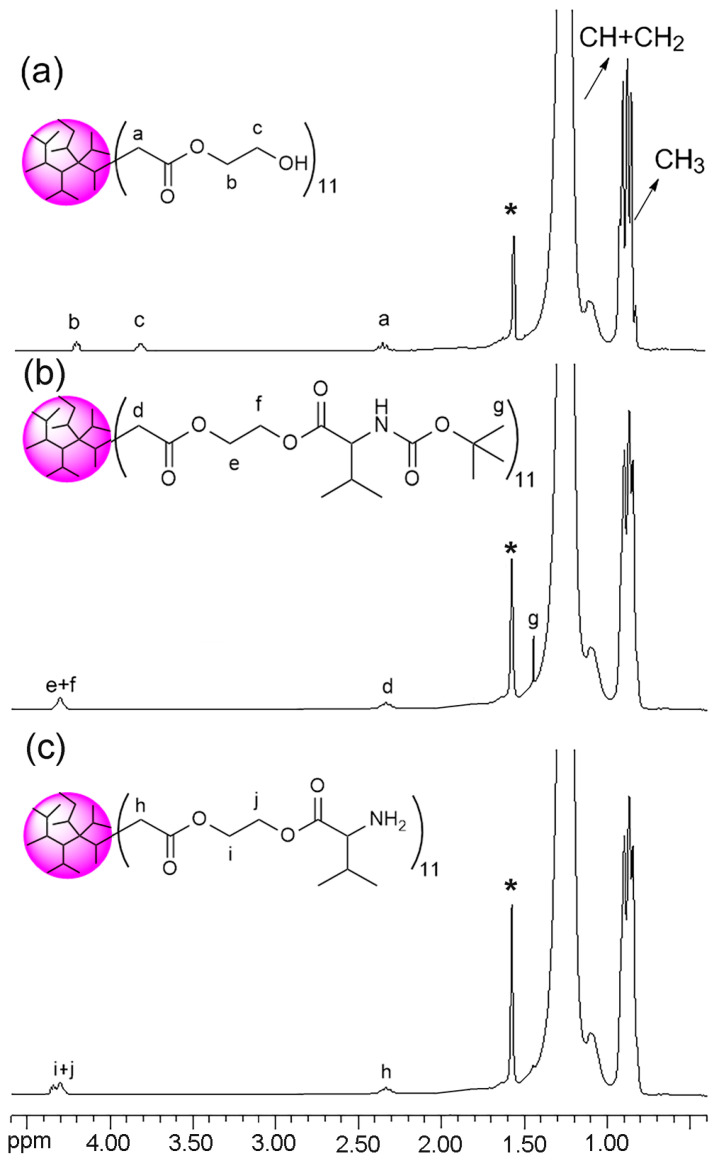
^1^H NMR spectra of DPE–(OH)_11_ (**a**), DPE–(BOC)_11_ (**b**), and DPE–(NH_2_)_11_ (**c**) (* water peak). (**a**–**c**) explanations.

As shown in Scheme 1, DPE-(PBLG)_n_ copolymers were prepared by palladium-catalyzed copolymerization of ethylene with polar comonomer in tandem with ring-opening polymerization (ROP) of BLG-NCA. Based on our previous work [11,24], the DPE containing peripheral multi-hydroxyl groups (DPE–(OH)_n_) was prepared by copolymerization of ethylene and HEA-TMS (2-hydroxyethyl acrylate with a protected hydroxyl by trimethylsilyl (TMS)) using a classic Brookhart-type palladium catalyst at 35 °C and low ethylene pressure of 0.1 atm after full deprotection of TMS using by tetrabutylammonium fluoride (TBAF). Because of dendritic architecture, the absolute number-average molecular weight (*M_n_*) of the obtained DPE–(OH)_n_ was 95 kg/mol with a polydispersity index (PDI) of 1.38 determined by GPC-LS using a light scattering detector. ^1^H NMR analysis (Figure 1) shows that the branching density of DPE–(OH)_n_ is 102/1000 C, and the n value (the average number of the terminated –OH per each molecule) was ~11 according to the incorporation of hydroxyl groups (0.32 mol%) (see experiment). Therefore, the obtained hydroxyl-functionalized DPE is a DPE–(OH)_11_ with *M_n_* of 95 kg/mol and PDI of 1.38. 

The terminated hydroxyl groups of DPE were converted into the amino groups by end-capping DPE–(OH)_11_ with N-*tert*-butoxycarbonyl-L-valine (BOC-L-valine) and deprotecting BOC group [24]. To improve efficiency of conversion, the excess of small molecule agents was used in the reaction. At same time, the easy solubility of the DPE in solvent and the exposition of hydroxyl groups on the periphery of DPE also facilitated quantitative conversion. The full conversion from the hydroxyl to the amine groups is confirmed by the disappearance of a characteristic triplet peak (**c**) (Figure 1a) at 3.72 ppm (–CH_2_OH) and the presence of methylene signal linked to ester (CH_2_–COO) (**e**+**f**) at 4.20 ppm (Figure 1b). Complete deprotection of BOC group was also proved by the disappearance of methyl signal (**g**) on the COOC(CH_3_)_3_ group (1.44 ppm) in the ^1^H NMR spectrum (Figure 1c).

### 3.2. Synthesis and Characterization of DPE-(PBLG)_n_ Copolymers

The amino-functionalized DPE–(NH_2_)_11_ was used a macroinitiator to initiate the ROP of BLG-NCA in CH_2_Cl_2_. Three samples of DPE-(PBLG)_11_ with a same DPE core but different PBLG arm lengths were synthesized (see Table 1) by changing BLG-NCA monomer addition. No weight loss was found by extracting copolymer products using petroleum ether to removal macroinitiator residuals, suggesting complete initiation of amino because of excellent solubility of the DPE in solvent. Attempts to determine copolymer molecular weights by GPC using THF or CHCl_3_ as eluent were unsuccessful. Overlapped elution traces with solvent were observed, which was ascribed to the strong interactions between the polypeptide-based polymers and GPC columns [38,40]. DMF in the presence of LiBr is a good eluent for GPC determination of PBLG homopolymer, but it is unsuitable for DPE-(PBLG)_n_ copolymers because self-assembly of DPE-(PBLG)_n_ is expected to take place in selective DMF solvent. 

**Figure 2:**
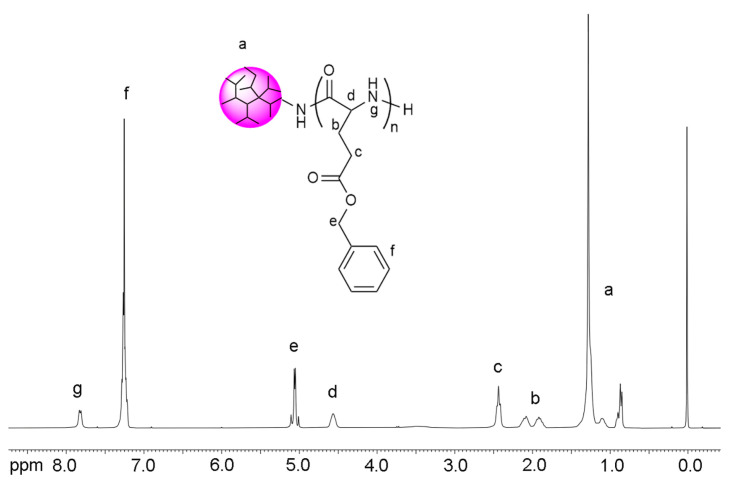
^1^H NMR spectrum of DPE-(PBLG)_11_.

It is reported that the primary anime-initiated ROP of *N*-carboxyanhydrides is a controlled reaction to afford polypeptides with narrow PDIs (PDI = 1.2–1.4) [40]. Therefore, it is expected that the DPE-(PBLG)_11_ copolymer has same PBLG arm length [46,47]. ^1^H NMR analysis was performed to characterize copolymer chemical structure and molecular weight. As shown in Figure 2, characteristic peaks of PBLG building blocks are clearly observed at 5.06 (peak **e**) and 7.81 ppm (peak **g**), indicating successful copolymerization. Based on the integral ratios of characteristic peaks (peak **e** vs. peak **a** for DPE), the degree of polymerizations (DPs) of γ-benzyl-L-glutamate are calculated to be 23, 31, and 45, respectively. Therefore, number-average molecular weights of three copolymers are also calculated and listed in Table 1. 

The thermal analyses of the obtained DPE-(PBLG)_11_ copolymer samples were performed using differential scanning calorimetry (DSC). In the second heating run, DPE-(PBLG)_11_ copolymers did not show any endothermic peaks. Firstly, the PE building block did not display the endothermic peak because of its dendritic structure. Secondly, PBLG segment also did not exhibit the liquid crystalline phase transition temperature, which was previously observed at 114–117 °C for some PBLG block copolymers [48,49,50]. Possibly, the unique core-shell structure of DPE-(PBLG)_11_ copolymer samples led to the disappearance of the liquid crystalline phase transition temperature of the PBLG segment.

**Figure 3:**
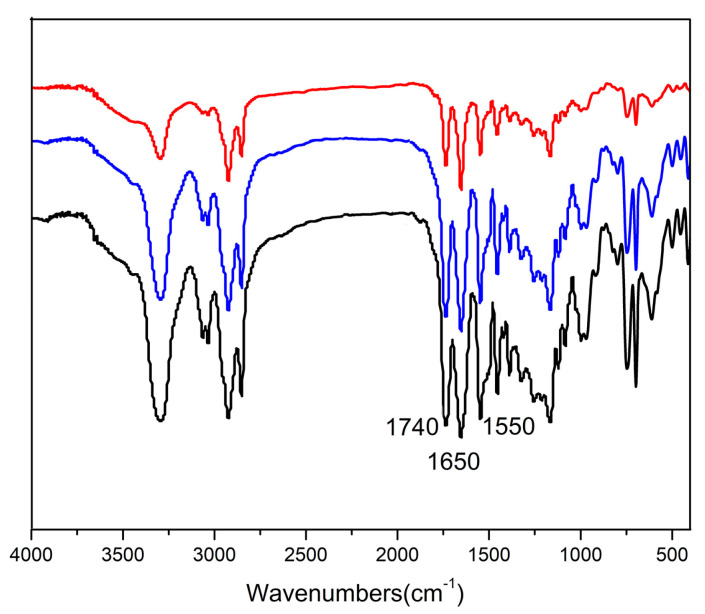
FTIR spectra of DPE-(PBLG)_11_ copolymer samples. DPE-(PBLG_23_)_11_ (red), DPE-(PBLG_31_)_11_ (blue), DPE-(PBLG_45_)_11_ (black).

PBLG can display three secondary structures including α-helix, β-sheet, and random coil conformations, which are determined by PBLG chain length [51]. FTIR analysis was used to characterize the secondary conformation structures of PBLG arms of DPE-(PBLG)_11_ copolymers. As shown in Figure 3, characteristic vibrational peaks (amide I band at 1650 cm^−1^, amide II band at 1550 cm^−1^) are clearly observed and no vibrational peaks of β-sheet conformation at ~1630 and 1530 cm^−1^ appear, indicating that three DPE-(PBLG)_11_ samples assume α-helix conformation. Therefore, the obtained DPE-(PBLG)_11_ copolymers have a unique architecture with a dendritic and coil core and multiple rod and helix arms. 

### 3.3. Gelation of DPE-(PBLG)_11_ Copolymer

It is known that PBLG homopeptide can form thermoreversible gels in toluene by hydrogen bonding interactions, while PBLG-based block copolymers also show thermoreversible gelation by a nanoribbon mechanism driven by π–π stacking interactions [52,53,54]. Although previous studies have shown that the architecture and chemical structure of PBLG-based copolymers have an important influence on gelation [38,52,53,54], copolymers with a dendritic and coil core and multiple PBLG arms have never been reported on or used to study the gelation behavior. Herein, DPE-(PBLG)_11_ copolymers were used to study their gelation in toluene and illuminate the effect of copolymer architecture.

**Figure 4:**
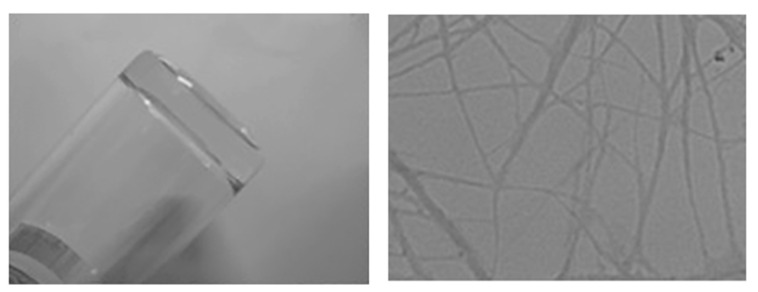
Gel image (**left**) generated from DPE-(PBLG_45_)_11_ in toluene and TEM photograph (**right**) of gel.

Three DPE-(PBLG)_11_ copolymers were soluble in hot toluene (90 °C) because of excellent solubility of DPE. When the solution temperature decreased below 70 °C, three organogels were formed. When organogels was heated up to >70 °C, the toluene solution was generated again (Figure 4, left). Therefore, DPE-(PBLG)_11_ copolymers showed thermoreversible gelation. The apparent critical gelation concentrations (*C*_gel_) and the critical gelation temperatures (*T*_gel_) of copolymers in toluene were further determined by the test tube inversion method and listed in Table 2. 

Previous literature has reported that PBLG homopolymer had a low *C*_gel_ of ~1.7% and low *T*_gel_ of 33 °C while PBLG block copolymers had a low *C*_gel_ of 0.2~2.0% and increasing *T*_gel_ of 40 °C~50 °C [38,52]. In our systems, fully stagnant organogels were only formed at high concentrations (*C*_gel_) of 10.5, 9.4, and 8.7 wt% for three samples DPE-(PBLG_23_)_11_, DPE-(PBLG_31_)_11_, and DPE-(PBLG_45_)_11_, respectively. Furthermore, high gel-sol phase transition temperatures (*T*_gel_) of 65 °C~70 °C were also determined. Obviously, high *C*_gel_ and *T*_gel_ are closely related to copolymer architecture of DPE-(PBLG)_11_. Dong previously reported dendron-like PBLG/linear poly(e-caprolactone)/dendron-like PBLG triblock copolymers had a high *C*_gel_ of 8 wt% [40]. Nandi and Banerjee found that the *T*_gel_ of dendritic peptide increased with increasing *C*_gel_, and reached 60 °C~160 °C in 1,2-dichlorobenzene [55]. Therefore, high *C*_gel_ and *T*_gel_ of DPE-(PBLG)_11_ copolymers are reasonably ascribed to the unique copolymer architecture with a dendritic PE core and multiple PBLG arms. Like PBLG based block copolymer, the chain length of the PBLG arm has a remarkable effect on the gelation of the DPE-(PBLG)_11_ copolymers. As shown in Table 2, the *C*_gel_ decreases but the *T*_gel_ increases with increasing PBLG length. The dependence of *C*_gel_ on the PBLG chain length strongly suggests that the driving force of the self-assembly is mainly originated from the π–π interaction between the different PBLG helices [40,55].

**Figure 5:**
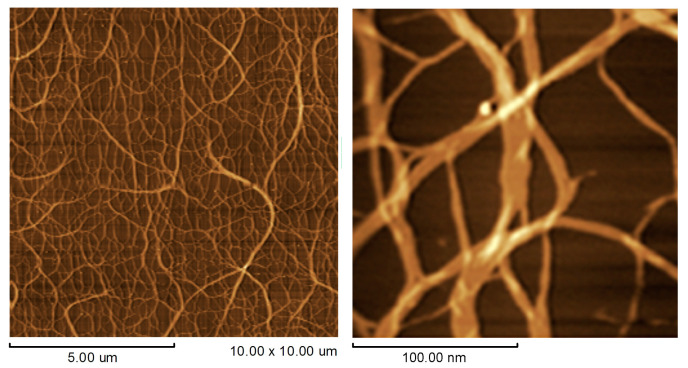
AFM image and magnified AFM image of gel generated from DPE-(PBLG_45_)_11_ in toluene.

To directly visualize the self-assembled nanostructures, transmission electron microscopy (TEM) and atomic force microscopy (AFM) were used to characterize gel sample. As shown in Figure 4, TEM image shows the assembly of DPE-(PBLG_45_)_11_ in a network of fibrous structure, and their average width was about 20 ± 5 nm. AFM images (Figure 5) also reveal a fibrillar network, and deep analysis of the AFM height profiles of DPE-(PBLG_45_)_11_ shows a height of ~2 nm, and a width of ~20 nm. Therefore, TEM and AFM clearly proved that the fibril morphology is not cylindrical but displays twisted flat ribbons. It is worthwhile that the self-assembled fibers formed by DPE-(PBLG_45_)_11_ are more heterogeneous than previously observed fibers formed by PBLG homopolymer and block copolymers [36,40]. For PBLG homopolymer and block copolymers, self-assembled nanostructures usually show intertwined structure (physical entanglement) among ribbons. In addition to physical entanglement, ramified structures are also observed in AFM macrograph (Figure 5), which is generated from of a DPE core (chemical crosslinking). Generally, the DPE core causes heterogeneous and ramified fibers.

**Figure 6:**
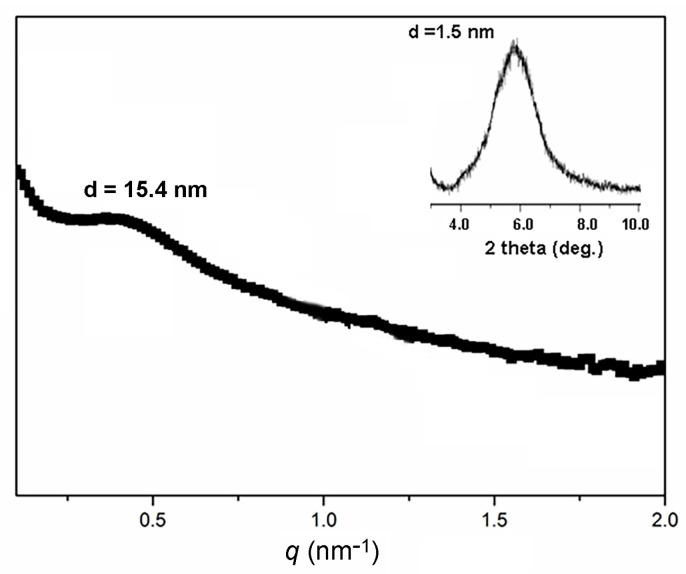
SAXS and WAXD (inset) profiles of the dried gel generated from DPE-(PBLG_45_)_11_.

Small-angle X-ray scattering (SAXS) detection on the dried organogel was also performed, and the data for the dried DPE-(PBLG_45_)_11_ toluene gel is shown in Figure 6. The ribbon width is revealed by a diffraction peak at 15.4 nm, which is basically consistent with TEM and AFM observations. Wide angle X-ray diffraction (WAXD) of the dried organogel of DPE-(PBLG_45_)_11_ also shows a diffraction peak at 1.5 nm, which corresponds to the distance between PBLG helices (inset of Figure 6). Therefore, the morphology of the fibers is a twisted flat ribbon formed by a 1-D monolayer stacking. In general, the experimental results in this work verify that the nanoribbon mechanism for the self-assembly of PBLG based block copolymers proposed by Manner also applies to DPE-(PBLG)_11_ copolymers with a dendritic PE and helical PBLGs arms [43]. We can reasonably imagine that DPE-PBLGs copolymers self-assemble in an anti-parallel fashion because of bulky dendritic PE, and not all PBLG arms get involved in self-assembly, which is responsible for high *C*_gel_ [52]. 

## 4. Conclusions

In conclusion, we have successfully synthesized coil–rod DPE-(PBLG)_11_ copolymers by combining palladium-catalyzed copolymerization of ethylene with HEA-TMS comonomer and ROP of BLG-NCA. The unique structure of copolymers is characterized to a DPE core and multiple helical PBLG arms. DPE-(PBLG)_11_ copolymers formed thermoreversible gels in toluene. The dendritic topology of DPE core increases the critical gelation concentrations and the critical gelation temperatures, while increasing PBLG chain length leads to a decrease in the critical gelation concentration. The self-assembled structure of gels is twisted flat ribbons formed through a self-assembled nanoribbon mechanism. The self-assembled fibers formed by DPE-(PBLG_45_)_11_ are more heterogeneous and ramified than previously observed fibers formed by PBLG homopolymer and block copolymers because of the DPE core. Such a self-assembly of coil DPE-rod PBLG copolymer is interesting, which shows a potential application in the field of biomaterial.

## Figures and Tables

**Table 1:** Synthesis and characterization of DPE-(PBLG)_11_ copolymers ^a^.

Entry	Polymer ^b^	NCA/DPE ^b^	Conversion (%)	*M_n_*_-NMR_^c^ (kg/mol)	*M_n_*_-PBLG_^c^ (kg/mol)	DP ^d^
1	DPE-(NH_2_)_11_	0	–	95.0	–	–
2	DPE-(PBLG_23_)_11_	400	63.2	161.0	6.0	23
3	DPE-(PBLG_31_)_11_	550	62.0	181.9	7.9	31
4	DPE-(PBLG_45_)_11_	800	61.9	224.8	11.8	45

^a^ Conditions: 25 °C, 72 h, 5 mL CH_2_Cl_2_, 0.2 g DPE-(NH_2_)_11_; ^b^ feeding ratio of BLG-NCA to DPE-(NH_2_)_11_; ^c^ Determined by ^1^H NMR spectroscopy.; ^d^ Degree of polymerization of BLG-NCA monomer calculated by *M_n_* of PBLG chain.

**Table 2:** Organogel parameters for DPE-(PBLG)_11_ copolymers.

Entry	Copolymer	*N*_PBLG_	*C*_gel_ ^a^ (wt%)	*T*_gel_ ^a^ (°C)
1	DPE-(PBLG_23_)_11_	23	10.5	65
2	DPE-(PBLG_31_)_11_	31	9.4	68
3	DPE-(PBLG_45_)_11_	45	8.7	70

^a^ Determined by the test tube inversion method.

## Data Availability

The data presented in this study are available on request from the corresponding author.
